# Antioxidants Supplementation in Elderly Cardiovascular Patients

**DOI:** 10.1155/2013/408260

**Published:** 2013-12-29

**Authors:** Matilde Otero-Losada, Susana Vila, F. Azzato, José Milei

**Affiliations:** Instituto de Investigaciones Cardiológicas (ININCA), Universidad de Buenos Aires (UBA), Consejo Nacional de Investigaciones Científicas y Técnicas (CONICET), M.T. de Alvear 2270 (C1122AAJ), Buenos Aires, Argentina

## Abstract

Supplementation with antioxidants and its benefit-risk relationship have been largely discussed in the elderly population. We evaluated whether antioxidants supplementation improved the biochemical profile associated with oxidative metabolism in elderly cardiovascular patients. Patients (*n* = 112) received daily supplementation with **α**-TP 400 mg, beta-carotene 40 mg, and vitamin C 1000 mg for 2 months (treatment). Plasma concentrations of alpha-tocopherol (**α**-TP), **β**-carotene (**β**C), ubiquinol-10 (QH-10), glutathione, and thiobarbituric acid reactive substances (TBARS) were determined before and after treatment. Response to treatment was dependent on pretreatment **α**-TP and **β**C levels. Increase in **α**-TP and **β**C levels was observed only in patients with basal levels <18 **μ**M for **α**-TP (*P* < 0.01) and <0.30 **μ**M for **β**C (*P* < 0.02). Ubiquinol-10, glutathione, and TBARS were unaffected by treatment: QH-10 (+57%, *F*_1,110_ = 3.611, *P* < 0.06, and N.S.), glutathione (+21%, *F*_1,110_ = 2.92, *P* < 0.09, and N.S.), and TBARS (−29%, *F*_1,110_ = 2.26, *P* < 0.14, and N.S.). Treatment reduced oxidative metabolism: 5.3% versus 14.6% basal value (*F*_1,110_ = 9.21, *P* < 0.0003). Basal TBARS/**α**-TP ratio was higher in smokers compared to nonsmokers: 0.11 ± 0.02 versus 0.06 ± 0.01 (*F*_32,80_ = 1.63, *P* < 0.04). Response to antioxidant supplementation was dependent on basal plasma levels of **α**-TP and **β**C. Smoking status was strongly associated with atherosclerotic cardiovascular disease and high TBARS/**α**-TP ratio (lipid peroxidation).

## 1. Introduction

Atherosclerotic cardiovascular diseases are a major cause of mortality and morbidity in the general population [1].

Numerous studies have focused on the utility of antioxidant supplementation in the treatment of cardiovascular diseases [2]. Yet, whether antioxidant supplementation has any preventive and/or therapeutic value in cardiovascular pathology is still a matter of debate for evidence is inconclusive [3–9]. Observational studies of vitamins C and E, the most prevalent natural antioxidant vitamins, suggest that supplemental use of these vitamins may lower the risk for coronary events [10]. High doses of antioxidants may pose risk due to adverse effects [11]. Advertising and marketing encourage consumption of vitamins supplements regardless of proper indication and supplements are ready available on-the-counter for self-medication. The estimated prevalence of dietary-supplement use among US adults was reported to be 73% not long ago [9]. In some populations, supplements are consumed to enhance general wellbeing following the advice of friends and magazines [12].

Oxidative stress results from the imbalance between oxidative metabolism and antioxidant activity and is involved in the pathogenesis of atherosclerotic cardiovascular disease (ACVD). Reactive oxygen species (ROS) are by-products of aerobic metabolism that are tightly controlled by antioxidants. Recently the function of ROS in cardiovascular pathology has been reviewed [13].

Antioxidants administration has proved to exert protection against injury in basic research studies [14–17] and imbalance of the antioxidant-oxidant ratio has been reported in experimental models of disease [18–20].

The aim of this study was to evaluate whether supplementation with antioxidants effectively modified the biochemical profile associated with oxidative metabolism in elderly patients undertaking periodical cardiovascular check. Vitamin C (ascorbic acid), vitamin E (*α*-tocopherol), and *β*-carotene are considered important antioxidants in humans and were tested in this study [21].

## 2. Materials and Methods

### 2.1. Design

One-hundred twelve outpatients undertaking periodical cardiovascular checks (51 men, 61 women, 69 ± 5 years, and living in Buenos Aires city) were selected according to the following criteria. Inclusion criteria are age (≥65 years), consumers of a varied diet as recorded in a previous nutritional interview. Exclusion criteria are heavy alcohol drinkers; patients consuming vitamin supplements; vegetarians, vegans, or followers of any restricted diet; patients recovering from an illness, surgery, or infectious process; patients with cerebrovascular events, for example, brain ischemia or stroke; and patients taking medications other than what is indicated (see 2nd paragraph below).

The patients were assigned to receive daily supplementation with *α*-TP 400 mg, beta-carotene 40 mg, and vitamin C 1000 mg with dinner for 2 months (end of the study) [22]. This administration schedule was considered appropriate and safe according to our team of nutritionists. A healthy diet was designed by one of the authors of this paper (Susana Vila, medical nutritionist) and the patients signed a written commitment to follow up the diet through the course of the study. At the end of the supplementation treatment data were validated according with a personal nutritional questionnaire completed by the patients on a daily basis.

Four groups of patients were found among those enrolled in this study: “Smoker” smoked more than five cigarettes/day for at least 1 year or after cessation at least 3 months before the beginning of the study; “atherosclerotic cardiovascular disease” (ACVD) had one or more of the following: angina, myocardial infarction, intermittent claudication of lower extremities, previous history of bypass surgery, or angioplasty. “Sedentary” regularly exercised for less than 3 hs/week. “Hypertensive” had diastolic/systolic blood pressure over 140/90 mmHg when sitting over three measurements at three consecutive visits. The patients went on taking their respective medication (ACVD: beta-blockers, aspirin, and statins; hypertensive: angiotensin converting enzyme inhibitors, angiotensin receptor blockers, or calcium channel blockers with or without diuretic thiazide) during the study.

The participants signed a written informed consent at the beginning of the study which was conducted in accordance with the Declaration of Helsinki (1964).

### 2.2. Laboratory Analysis

Plasma concentrations of alpha-tocopherol (*α*-TP), *β*-carotene (*β*C), ubiquinol-10 (QH-10) (HPLC-UV-ED), glutathione (enzymatic assay) [23], and thiobarbituric acid reactive substances (TBARS) (fluorimetry of lipid oxidation products after plasma incubation in the appropriate media, excitation 515 nm/555 nm emission) [24] were determined before (basal) and after antioxidant supplementation (treatment). Percentage of oxidation was calculated as %TBARS in incubated samples/%TBARS in nonincubated samples. HPLC isocratic reverse phase separations were performed using Supelcosil 3 *μ*m LC-8DB column (4.6 mm × 3.3 cm) (Supelco, Bellefonte, USA), LiClO_4_ 2 × 10^4^ *μ*M in methanol : H_2_O (99 : 1, v/v) as mobile phase (1 mL/min flow rate). EC detection: a BAS LC4C amperometric detector with glassy-carbon working electrode (Bioanalytical Systems Inc., West Lafayette, IN, USA) is set at +0.6V UV detection: a Waters 460 Tunable absorbance detector was used (Millipore Corp., Milford, USA) working at *λ* = 275 nm.

Data was submitted to MANOVA followed by multidimensional scaling with cluster analysis or bivariate correlation analyses (Pearson's product moment correlation coefficient) in order to evaluate the main effects of treatment, data distribution, and the degree of association between variables [25]. Conventionally, the level of statistical significance was set at *P* < 0.05 (SPSS 15.0 software, SPSS Inc., Chicago, USA).

## 3. Results and Discussion

The sample of patients comprised smokers (29%), hypertensives (18%), sedentary subjects (63%), and patients with ACVD (23%).

The following factors were not related to interindividual variation in basal levels of antioxidants or TBARS: age (*F*_4,95_ = 1.34, *P* < 0.26, and N.S.), diabetes (*F*_4,95_ = 1.41, *P* < 0.23, and N.S.), ACVD (*F*_4,95_ = 1.45, and *P* < 0.22, N.S.), or sedentarism (*F*_4,95_ = 0.78, and *P* < 0.54, N.S.).

Smoking status was strongly associated with atherosclerotic cardiovascular disease (ACVD): 42% of smokers had ACVD compared with 16% of ACVD cases observed in nonsmokers (correlation coefficient = 0.87, *P* < 0.0001). This association was not surprising.

Basal TBARS/*α*-TP ratio (prooxidant/antioxidant imbalance) was higher in smokers compared to nonsmokers: 0.11 ± 0.02 versus 0.06 ± 0.01, respectively (*F*_32,80_ = 1.63, *P* < 0.04) (Table 1). Alpha-TP, *β*C, glutathione, and ubiquinol-10 levels were dissociated from smoking condition (yet an overall trend to lower antioxidant levels was observed in smokers).

Plasma levels of either *α*-TP or *β*C were not affected by treatment according to average values of the entire sample of patients. However cluster analysis split the sample into two categories of patients based on pretreatment *α*-TP or *β*C levels and the plasma levels varied accordingly. Increases in *α*-TP or *β*C levels were observed only in patients with basal levels below either 18 *μ*M for *α*-TP (*P* < 0.01) or 0.30 *μ*M for *β*C (*P* < 0.02) (Table 2). The value of 18 *μ*M for *α*-tocopherol has been elsewhere considered as the cut point between low and normal *α*-TP plasma concentration ranges [26, 27].

Ubiquinol-10, glutathione, and TBARS levels did not show significant changes following supplementation irrespective of *α*-TP and *β*C basal levels: QH-10 (57% increase, *F*_1,110_ = 3.611, *P* < 0.06, and N.S.), glutathione (21% increase, *F*_1,110_ = 2.92, *P* < 0.09, and N.S.), TBARS (29% decrease, *F*_1,110_ = 2.26, *P* < 0.14, and N.S.) (Table 2).

Antioxidants supplementation reduced the percentage of oxidation to 5.26 ± 0.42% compared with 14.60 ± 2.19% found at the beginning of the study (*F*_1,110_ = 9.21, *P* < 0.0003). Data analysis revealed that the overall decrease in the percentage of lipid oxidation following antioxidant supplementation was mainly attributable to the change observed in patients with basal *α*-TP levels <18 *μ*M (*P* < 0.0003). Only a trend (*P* < 0.059, N.S.) was found for the association between the change in lipid oxidation and *β*C baseline levels. This observation may drive the conclusion that antioxidant supplementation at the doses administered in the present study, similar to those routinely found in over-the-counter multivitamin supplements, may have some benefits irrespective of the starting *β*C plasma levels.

Overall the subjects did not report any beneficial effects after antioxidants' supplementation except for subjective observations such as “feeling more vital” or a feeling of “general wellbeing”; however, a placebo effect should not be ruled out.

Association of smoking with high oxidant/antioxidant ratio has been reported previously [28, 29].

Only a few of several meta-analyses of the findings of clinical trials using antioxidant supplementation are mentioned below [30–34]. Several cohort studies suggested reduced cardiovascular risk in persons taking vitamin E supplements. However, randomized clinical trials of vitamin E did not show any benefit of vitamin E supplementation in terms of prevention of coronary heart disease and death [30]. Identical rates of cardiovascular death were found for the placebo and vitamin groups, though a small but significant increase in CVD was found to be associated with *β*-carotene supplementation in a meta-analysis that included 7 trials using vitamin E in >81,000 patients and 8 *β*-carotene trials with >138,000 patients [31]. A meta-analysis of 19 clinical trials comprising a total of 135967 participants revealed that supplementation with high doses (16.5 to 2000 IU/d) of vitamin E may cause a slight increase in mortality. A further meta-analysis of the same 19 clinical trials with the inclusion of 10 additional trials (2495 participants, vitamin E doses 136 to 5000 IU/d) was later performed and yielded contradictory results. While the former results were confirmed, the results also indicated that the increased mortality odds ratio was not related to supplementation with high doses of vitamin E in some trials [33].

As a part of a European multicentre project, a study (400 healthy volunteers, 25–45 years) reported that supplementation with alpha-tocopherol and/or carotenoids increased respective serum levels and that no significant side effects (except for carotenodermia) or changes in biochemical or haematological indices had been observed [34].

Oxidative stress has been implicated in pathophysiology of aging and age-associated disease and antioxidants supplementation has become a practice for prevention of atherosclerosis and cardiovascular disease [35]. Clinical studies have not demonstrated a benefit of vitamin E in the primary and secondary prevention of cardiovascular disease. Vitamin E supplementation was associated with increased mortality, heart failure, and hemorrhagic stroke [1]. The American Heart Association does not support the use of vitamin E supplements to prevent cardiovascular disease and recommends the consumption of foods rich in antioxidant vitamins and minerals [1].

Supplementation is usually decided on the assumption that endogenous antioxidants' levels are below the accepted values sometimes underestimating that adverse effects may appear. Supplementation adds an extra burden to the liver and kidneys particularly in elderly patients. A simple laboratory analysis provides information on endogenous antioxidants levels and it may additionally help in reaching a more accurate diagnosis by ruling out (or not) hypothetical7 nutritional deficits.

Two major findings were observed in elderly cardiovascular patients in this study. The increase in plasma levels of *α*-TP or *β*C was dependent on the respective basal levels. Smoking status was strongly associated with atherosclerotic cardiovascular disease and high TBARS/*α*-TP ratio (lipid peroxidation).

Higher prevalence of ACVD found in smokers agrees with the idea of an oxidative pathogenic substrate in ACVD.

No differences were observed in *α*-TP, *β*C, glutathione, or ubiquinol-10 plasma levels between smokers and nonsmokers in this study in agreement with previous reports [36]. Yet, smoking was associated with higher TBARS/*α*-TP ratios suggesting an increase in lipid peroxidation relative to antioxidant activity. To our knowledge, this observation was not previously reported. Cigarette smoking is widely accepted to be a major cardiovascular risk factor.

Low plasma levels of antioxidants have been associated with endothelial dysfunction, the first step towards atherosclerosis [37, 38] and increased cardiovascular risk. In the present study we considered the relationship between ACVD and plasma levels of antioxidants. Our results do not support the association between ACVD and low serum concentrations of *α*-tocopherol, *β*-carotene, glutathione, or ubiquinol-10. The small number of ACVD patients in this study (*n* = 26) may partly account for this discrepancy. It is possible that some of these patients had increased consumption of vegetables and other sources of antioxidants after they suffered a major event of coronary or peripheral vascular disease as well. Other authors arrived to similar results and did not find differences in plasma *α*-TP levels but observed higher *α*/*γ*-tocopherol ratio in patients with coronary heart disease [39].

In this study hypertension, a known risk factor of ACVD, was not associated with differences in plasma antioxidants or TBARS concentrations.

## 4. Conclusions

The effectiveness of antioxidant supplementation to modify plasma biochemistry as a result of changes in oxidative metabolism was dependent on basal endogenous antioxidants levels. Present results suggest that awareness of basal plasma antioxidants levels might be advisable before starting supplementation with antioxidants in elderly cardiovascular patients, a population in which special precautions are recommended. Arguably excess antioxidant levels in tissues may lead to deleterious consequences [40–42]. However, no adverse effects were reported during the course of this study.

## Figures and Tables

**Table 1:** Plasma levels of antioxidants and oxidative stress' parameters at the beginning of the study.

	Smokers (*n* = 32)	Nonsmokers (*n* = 80)	Hypertensive (*n* = 20)	Sedentary (*n* = 71)	ACVD (*n* = 26)
*α*-TP	19.21 ± 2.73	22.15 ± 2.29	21.33 ± 3.15	21.98 ± 2.34	20.37 ± 2.44
*β*-Carotene	0.29 ± 0.03	0.34 ± 0.03	0.32 ± 0.05	0.33 ± 0.04	0.27 ± 0.05
Ubiquinol-10	0.22 ± 0.04	0.31 ± 0.05	0.26 ± 0.04	0.26 ± 0.03	0.25 ± 0.07
Glutathione	0.64 ± 0.04	0.73 ± 0.08	0.68 ± 0.06	0.69 ± 0.05	0.67 ± 0.08
TBARS	2.41 ± 0.63	1.31 ± 0.37	1.77 ± 0.36	1.56 ± 0.29	1.97 ± 0.31

TBARS/*α*-TP	0.13 ± 0.03^#^	0.06 ± 0.02	0.08 ± 0.02	0.08 ± 0.01	0.10 ± 0.17
Lipid oxidation (%)	18.71 ± 2.49*	12.61 ± 2.18	16.62 ± 1.98	15.31 ± 2.27	16.15 ± 2.07

Plasma concentration is expressed in *μ*M as mean value ± SEM; *α*-TP: *α*-tocopherol; TBARS: thiobarbituric acid reactive substances.

*P < 0.05, ^#^ *P* < 0.04 versus nonsmokers.

**Table 2:** Effect of antioxidant supplementation on biochemical profile related to oxidative metabolism.

	Plasma concentration (*μ*M)			Sig.
	Pretreatment	Posttreatment	Ratio (post-/pretreatment)	
*α*-TP	≥18	28.56 ± 2.60	1.07 ± 0.09	*P* < 0.01
	<18	25.76 ± 2.71	1.61 ± 0.11	
*β*C	≥0.30	0.39 ± 0.06	1.05 ± 0.17	*P* < 0.02
	<0.30	0.49 ± 0.17	2.94 ± 0.36	
Ubiquinol-10	0.27 ± 0.02	0.42 ± 0.14	1.57 ± 0.34	NS
Glutathione	0.70 ± 0.02	0.85 ± 0.16	1.21 ± 0.21	NS
TBARS	1.52 ± 0.08	1.07 ± 0.10	0.71 ± 0.09	NS
Lipid oxidation (%)	14.60 ± 2.19	5.26 ± 0.42	0.36 ± 0.08	*P* < 0.0003

*α*-TP: *α*-tocopherol; *β*C: *β*-carotene; TBARS: thiobarbituric acid reactive species. Values are expressed as mean ± SEM.
